# Numerical Analysis and Theoretical Study on the Interfacial Bonding Behavior of High-Strength Steel Stainless Wire Mesh-Reinforced ECC and Concrete

**DOI:** 10.3390/ma17235912

**Published:** 2024-12-03

**Authors:** Chao Li, Yao Zou, Ziyuan Li, Xuyan Zou, Ke Li, Juntao Zhu, Hongbo Xiao, Jianwei Fan

**Affiliations:** 1 Department of Civil Engineering, Zhengzhou University, Zhengzhou 450001, China; 2 Henan Provincial Government Offices Administration, Zhengzhou 450008, China; 3 Department of Civil Engineering, Zhengzhou Institute of Technology, Zhengzhou 450044, China; 4 Department of Civil Engineering, Henan Vocational College of Water Conservancy and Environment, Zhengzhou 450008, China

**Keywords:** high-strength steel stainless wire mesh-reinforced ECC, interfacial bonding properties, numerical analysis, effective anchorage length

## Abstract

In order to investigate the interfacial bonding properties of high-strength steel stainless wire mesh-reinforced ECC (HSSWM-ECC) and concrete, a finite element model was established for two types of interfaces based on experimental research. The results show that the failure modes observed in the 21 groups of simulations can be classified into three categories: debonding failure, ECC extrusion failure and concrete splitting failure. The failure mode was mainly affected by the type of interface. The effective anchorage length is inversely proportional to the strength of the concrete and proportional to the stiffness and thickness of the HSSWM-ECC. The capacity of the roughening interface is positively correlated with the concrete strength and bonding length, but negatively correlated with the interfacial width ratio. Increasing both the number and width of grooves within the effective range enhances the interfacial capacity, whereas higher concrete strengths tend to reduce it. Based on the above results, calculation models for the effective anchorage length and bearing capacity were established separately for the two types of interfaces. The theoretical model for the interfacial bonding property between HSSWM-ECC and concrete has been refined. These advancements establish a theoretical groundwork for the design of concrete structures strengthened with HSSWM-ECC.

## 1. Introduction

Engineered cementitious composites (ECCs) have excellent strain-hardening properties, crack dispersion capacity and high ductility; however, they have the shortcoming of insufficient tensile strength [1,2,3,4,5,6,7,8]. High-strength steel stainless wire mesh (HSSWM) has the potential to be a reinforcement for ECC [9,10,11,12]. Research has demonstrated that HSSWM exhibits excellent bonding compatibility with ECC due to the rough surface texture of the steel strands, which enhances the mechanical interlocking between the materials [4,13]. This strong interfacial bond enables the HSSWM-ECC composite to achieve an ultimate tensile stress exceeding 6.0 MPa and an ultimate tensile strain ranging from 3% to 5%. Additionally, HSSWM-ECC demonstrates strong crack-width control, as evidenced by tensile and bending tests showing a high density of fine cracks alongside two or more wider primary cracks [14,15]. In addition, the bearing capacity and crack resistance of RC beams and columns strengthened with HSSWM-ECC can be significantly improved [16,17]. The maximum crack width of RC short columns strengthened with HSSWM-ECC is limited to 0.25 mm under peak load. Both the cracking load and peak load for these strengthened columns are approximately 100% higher than those of unreinforced columns. Additionally, crack propagation in the original RC beam is effectively mitigated, resulting in a significant reduction in crack width. This enhancement demonstrates the effectiveness of HSSWM-ECC in improving the structural performance and durability of RC elements.

Robust interfacial bonding between the strengthening materials and concrete is essential for achieving effective reinforcement [18], and plenty of scholars have conducted research into the bonding properties between strengthened materials and concrete. Carloni et al. [19] addressed the bond behavior of polyparaphenylene benzobisoxazole (PBO) fiber-reinforced cementitious matrix (FRCM) composites and concrete via a single-lap direct-shear test. The results indicate that the peak load of specimens with two fiber mesh layers is double the peak load of tests with one fiber mesh layer. Guo et al. [20] investigated the interfacial bonding properties between a polymer cement mortar carbon fiber mesh reinforcement layer and concrete, and proposed methods to ensure the bonding property of the interface. Cao et al. [21] demonstrated that the bonding property is significantly affected by the strength of the concrete through double shear tests of externally bonded carbon fiber-reinforced polymer (CFRP) panels and concrete specimens. Pan et al. [22] studied the interfacial bonding properties between CFRP chains and concrete, and established an interfacial bond-slip model between CFRP chains and concrete. Wang [23] has demonstrated that increasing the strength and interfacial roughness of existing concrete can improve the interfacial bond strength between ECC and concrete. Cai et al. [24] has proven that different interfacial treatment methods can improve the interfacial bonding properties of specimens to varying degrees. Zhang et al. [25] conducted research into the interfacial bonding properties between concrete and HSSWM-ECC. Test results showed that increasing the strength of the concrete as well as the interfacial bonding length and roughness can improve the interfacial bearing capacity. A calculation model for the interfacial bearing capacity of HSSWM-ECC and concrete was established based on the test results.

However, complete data on the failure process was unable to be obtained in the experimental study due to the sudden failure of the specimens. Brittle failure occurs easily when the anchorage length is less than 180 mm in the test. The final load and strain were unable to be obtained, leading to a small value for the peak load and slip. The theoretical results established thus need to be verified and improved. Therefore, this paper has explored the interfacial bonding properties of HSSWM-ECC and concrete by combining the preliminary test results and finite element simulation analysis. The failure mode, interfacial bearing capacity and interfacial stress development were analyzed, considering the influence of concrete strength, interfacial bond length and width and interfacial characteristics. A calculation model for effective anchorage length and bearing capacity was proposed based on a numerical analysis, and the theoretical model for interfacial bonding between HSSWM-ECC and concrete was enhanced. These developments provide a theoretical foundation for designing concrete structures strengthened with HSSWM-ECC.

## 2. Experimental Study

### 2.1. Experimental Design

**Figure 1:**
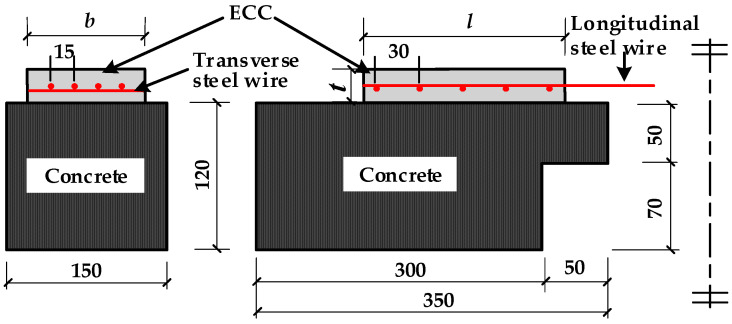
Geometry of specimen (Unit: mm).

**Figure 2:**
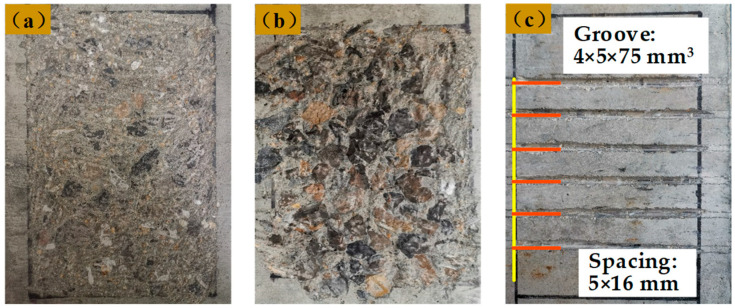
Processing modes of interface (**a**) Manual roughening; (**b**) High-pressure water flushing; (**c**) Mechanical grooving.

In the previous experimental study on the interfacial bonding properties of HSSWM-ECC and concrete [25], 9 groups of 27 beam-hinge tests [26] were designed and completed, and the main parameters are shown in Table 1. The specimen size is shown in Figure 1. The interface characteristics have an impact on the bonding behavior of materials [27]. In the test, three treatment methods were adopted for the concrete surface: manual roughening (R), high-pressure water flushing (F) and mechanical grooving (G). The concrete surfaces are shown in Figure 2. The characteristics of the groove include the width (4 mm), depth (5 mm) and adjacent groove spacing (16 mm). The roughness of the three types of interfaces were Rt = 1 mm, Rt = 2 mm and Rt = 1 mm, respectively.

**Figure 3:**
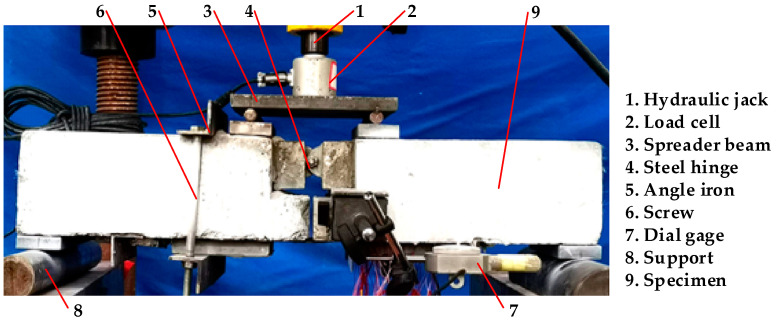
Loading device.

The test loading device is shown in Figure 3. A hydraulic jack (Guanhang, Hangzhou, Zhejiang, China) with an ultimate capacity of 100 kN was employed, and the compression load was applied to the specimen at a constant loading rate of 10 N/s. The non-measuring area was anchored with screw and angle bars to make the failure occur in the measuring area. The steel hinge in the midspan is defined as the loading end, and the support is defined as the free end. In order to measure the relative slip between the interface at the loading end, the dial indicator (Huoto, Yiwu, Zhejiang, China) with a magnetic support was adsorbed to the angled steel on the concrete.

### 2.2. Test Results

**Figure 4:**
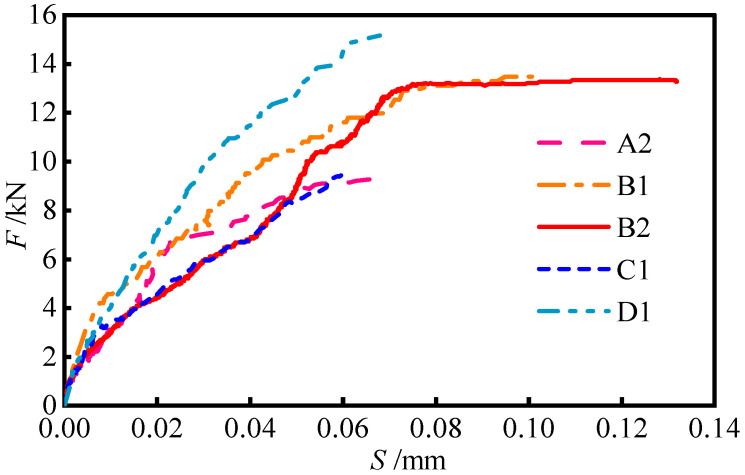
Interfacial load–slip curves of main specimens.

The test results are shown in Table 2. Three failure modes were found in the 27 specimens: the debonding failure of the interface (P), the breaking failure of the steel wires (R) and the splitting failure of the concrete (S). As shown in Table 2, increasing the bond length within the effective bonding range enhances the interfacial bearing capacity. Increasing the bonding width has no impact on the bearing capacity, whereas a higher concrete strength and interface roughness can improve the interfacial bearing capacity. The interfacial load–slip curves (F–S curves) are shown in Figure 4. The curves distinctly present two stages: a nonlinear ascending section and horizontal section.

At the initial stage of loading, the bonding interface is in the elastic stage. With increases in the load, the slope of the curve begins to decline, the interface bond stiffness decreases and the slip increases. For the specimen whose interface bonding length exceeds the effective anchoring length, the load tends to be stable after increasing to the peak value, but the slip continues to increase until failure. In addition, increasing the interface roughness can significantly improve the interface bonding stiffness. Changing the interface bonding length has little effect on the interface bonding stiffness, while increasing the interface bonding width decreases the interface bonding stiffness.

## 3. Establishment and Verification of FE Model

### 3.1. Material Model

The FE software DIANA (https://dianafea.com/, accessed on 13 January 2021) was adopted to explore the interfacial bonding property between the HSSWM-ECC and concrete. The concrete and ECC were set as hexahedral units (CHX60). The pad and steel hinge were set as hexahedral units (HX24L). The steel wires wrapped inside the ECC were set as an embedded steel bar unit. The exposed steel wires were set as an ordinary truss unit (L2TRU). The displacement interpolation function in the simulation calculation is shown in Equation (1):

(1) $$u_{x}\left(\xi \right)=a_{0}+a_{1}\xi$$

**Figure 5:**
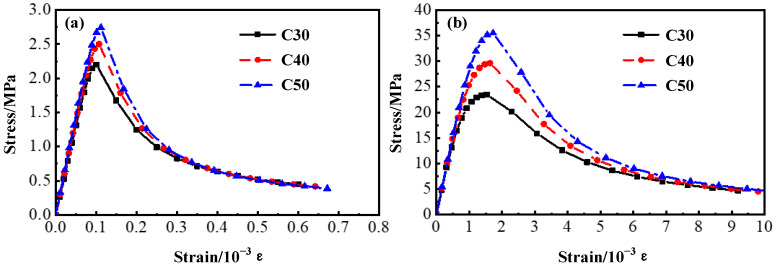
Constitutive models of concrete: (**a**) Tensile constitutive model of concrete; (**b**) Compressive constitutive model of concrete.

The concrete was simulated using the total strain crack model [28], while the crack was modeled using the rotating crack model [29]. The 28 day compressive strength of the C30, C40 and C50 concrete used in the test was 35.31 MPa, 46.03 MPa and 56.17 MPa, respectively. Constitutive parameters were determined through Table 3. The stress–strain curves of the concrete used in the test were calculated according to the curve model in the Code for Design of Concrete Structures, as shown in Figure 5.

**Figure 6:**
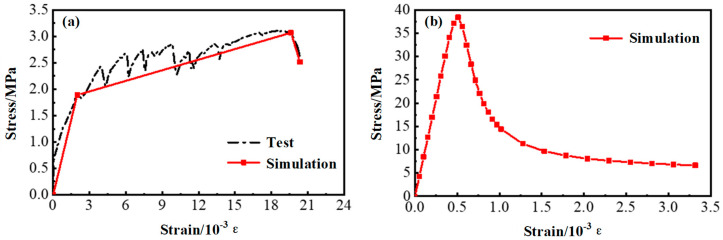
Constitutive models of ECC: (**a**) Tensile constitutive model of ECC; (**b**) Compressive constitutive model of ECC.

The ECC was simulated by adjusting the parameters based on the simulating method of concrete. The constitutive parameters of the ECC were determined according to Table 4. The tensile stress–strain relationship of the ECC is shown in Figure 6a. The expression of the tensile relationship is shown in Equation (2). The compressive stress–strain relationship is shown in Figure 6b, and the compressive relationship is shown in Equation (3).

(2) $$\sigma =\left\{\begin{array}{ll} E_{e}\varepsilon & \varepsilon \leq \varepsilon_{etc}\\ \frac{\sigma_{etu}-\sigma_{etc}}{\varepsilon_{etu}-\varepsilon_{etc}}(\varepsilon -\varepsilon_{etc})+\sigma_{etc} & \varepsilon_{etc}<\varepsilon \leq \varepsilon_{etp}\\ \frac{\sigma_{etp}-\sigma_{etu}}{\varepsilon_{etp}-\varepsilon_{etu}}(\varepsilon -\varepsilon_{etp})+\sigma_{etu} & \varepsilon_{etp}<\varepsilon \leq \varepsilon_{etu} \end{array}\right.$$

where *σ_etc_* and *ε_etc_* are the ECC cracking stress and strain, respectively; *σ_etp_* and *ε_etp_* are the peak tensile stress and strain of the ECC, respectively. *σ_etu_* and *ε_etu_* are the ECC tensile limit stress and strain, respectively. *E_e_* is the ECC elastic modulus. According to the test results, these values were as follows: *σ_etc_* = 1.81 MPa, *ε_etc_* = 0.014%, *σ_etp_* = 3.07 MPa, *ε_etp_* = 1.95%, *σ_etu_* = 0.05 MPa, *ε_etu_* = 2.3%, *E_e_* = 14,500 MPa.

(3) $$\sigma =\left\{\begin{array}{ll} \left[1.1\frac{\varepsilon }{\varepsilon_{ecp}}+0.5{\left(\frac{\varepsilon }{\varepsilon_{ecp}}\right)}^{5}-0.6{\left(\frac{\varepsilon }{\varepsilon_{ecp}}\right)}^{6}\right]\sigma_{ecp}\; & 0\leq \frac{\varepsilon }{\varepsilon_{ecp}}<1\\ (\frac{0.15{\left(\frac{\varepsilon }{\varepsilon_{ecp}}\right)}^{2}}{1-2\frac{\varepsilon }{\varepsilon_{ecp}}+1.15{\left(\frac{\varepsilon }{\varepsilon_{ecp}}\right)}^{2}})\sigma_{ecp} & \frac{\varepsilon }{\varepsilon_{ecp}}>1 \end{array}\right.$$

where *σ* and *ε* are the ECC compressive stress and strain, respectively; *σ_u_* and *ε_u_* are the peak compressive stress and compressive strain of the ECC, respectively. According to the test results, these values are as follows: *σ_u_* = 38.43 MPa and *ε_u_* = 0.52%.

**Figure 7:**
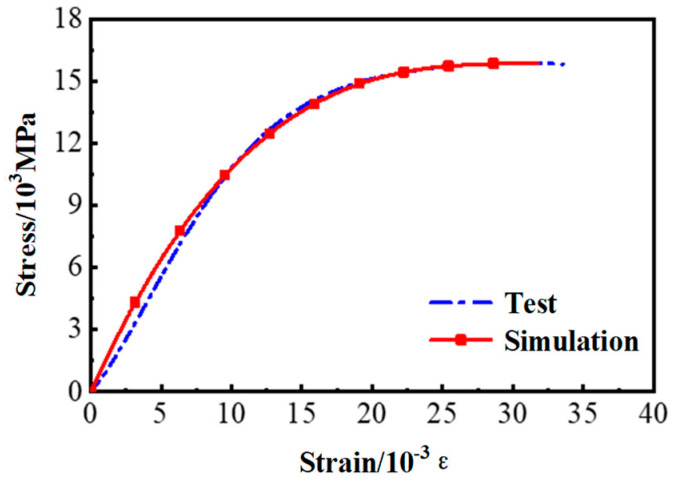
Constitutive model of steel wire.

The HSSWM was simplified as an isotropic material, and a nonlinear uniaxial model was used for simulations. The constitutive parameters of the HSSWM are shown in Table 5. The strain-hardening elastoplastic model was adopted, and Equation (4) was used as the objective function to fit the stress–strain curve of the HSSWM measured in the test. The fitting results are shown in Figure 7.

(4) $$\sigma =\sigma_{sp}\left[a{\left(\frac{\varepsilon }{\varepsilon_{sp}}\right)}^{3}+b{\left(\frac{\varepsilon }{\varepsilon_{sp}}\right)}^{2}+c\left(\frac{\varepsilon }{\varepsilon_{sp}}\right)\right]\;\;0\leq \varepsilon <\varepsilon_{sp}$$

where *σ_sp_* and *ε_sp_* represent the peak tensile strength and corresponding strain of the HSSWM, respectively. *a*, *b* and *c* are dimensionless coefficients obtained by fitting experimental data, and these values are shown in Table 5.

### 3.2. Bonding Interface Model

**Figure 8:**
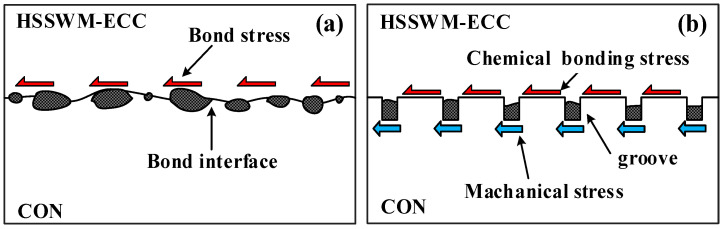
Mechanical model of bonding interfaces: (**a**) Interface I; (**b**) Interface II.

In view of the differences between the two interfaces, the interface formed by manual roughening and high-pressure water flushing is defined as Interface I and the interface formed by mechanical grooving is defined as Interface II. The mechanical models of the two bonding interfaces are shown in Figure 8. Different modeling methods were used to simulate the two interfaces.

**Figure 9:**
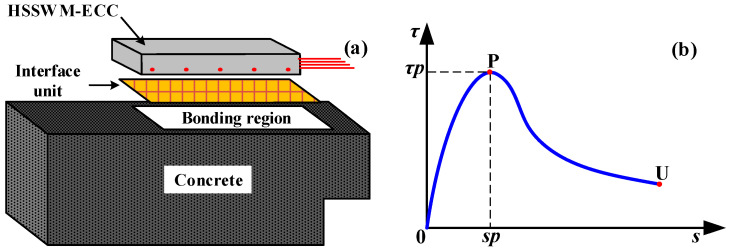
Model of Interface I: (**a**) Zero-thickness interface unit; (**b**) Local bond-slip model of the HSSWM-ECC and concrete.

**Figure 10:**
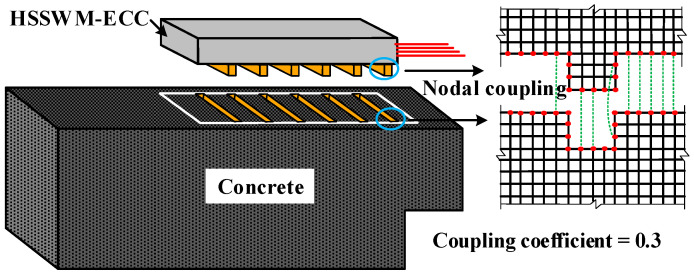
Model of Interface II.

A zero-thickness interface unit was inserted between the ECC and concrete to simulate Interface I, as shown in Figure 9a. The element type of the interface was CQ48I, whose tangential stiffness (*K_t_*) was taken as 2.5 × 10^10^ N/m, and the two normal stiffnesses (*K_v_*_1_ and *K_v_*_2_) were taken as 3.5 × 10^10^ N/m. The HSSWM-ECC and concrete interface local bond-slip model is shown in Figure 9b and Equation (5). Solid modeling of the groove was carried out to simulate Interface II, and the node at the contact position of the concrete and HSSWM-ECC was coupled. The coupling coefficient of 0.3 was assigned to the coupling node, as shown in Figure 10.

(5) $$\tau =\left\{\begin{array}{ll} \tau_{p}{\left(\frac{s}{s_{p}}\right)}^{0.75} & s\leq s_{p}\\ \tau_{p}e^{-{\left(\alpha ((s/s_{p})-1)\right)}^{2}} & s>s_{p} \end{array}\right.$$

where *τ* (MPa) is the bonding stress corresponding to the slip *s* (mm); *τ_p_* (MPa) is the peak bonding stress; *s_p_* (mm) is the corresponding slip; *α* is the coefficient shown in Equation (6).

(6a) $$\alpha =1/(\frac{G_{e}}{\tau_{p}s_{p}}-\frac{2}{3})$$

(6b) $$G_{e}=0.004175\sqrt{\frac{1.05-b/b_{c}}{0.05+b/b_{c}}}\beta_{a}f_{cu}l_{0}$$

where *G_e_* (N·mm^−1^) represents the interface strain energy; *f_cu_* (MPa) represents the cubic compressive strength of concrete; *β_a_* represents the influence coefficient of the interface treatment method. The *β_a_* of the manual roughening interface was equal to 1.0; the *β_a_* of the high-pressure water flushing interface was equal to 1.9; *l*_0_ is the dimensional parameter, and *l*_0_ = 1 mm.

### 3.3. Boundary and Meshing

**Figure 11:**
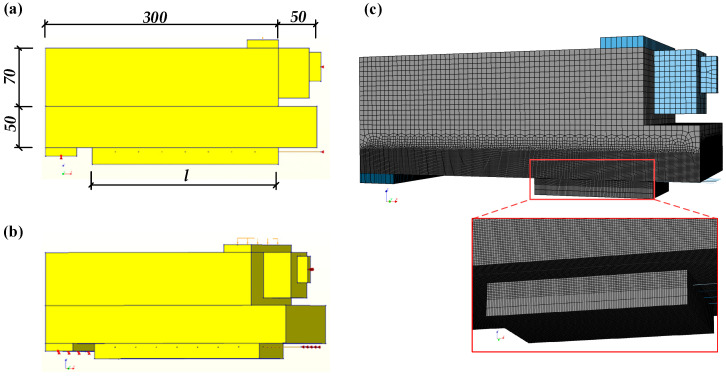
FE model of specimen: (**a**) Geometric view 1; (**b**) Geometric view 2; (**c**) meshing.

Full-size solid modeling was carried out to simulate half of the beam-hinge specimen, constraining the X-direction displacement between the center line of the steel hinge and the end of the midspan of the steel wire. The z displacement of the center of the bottom end of the concrete pad was also constrained. At the center line of the cushion block at the upper end of the concrete, Z-direction displacement was applied by displacement loading. The FE geometric model is shown in Figure 11. The model is divided into hexahedral grids by sweeping. A mesh size sensitivity analysis was performed for the finite element models. Locally fine meshes were used for the part close to the interface because the dominant damage was localized on the bonding interface. Specially, the size of the mesh element was set to 1 mm near the bonding interface, and the size was enlarged (2 mm–5 mm) at the position far away from the bonding interface. The mesh division of the finite element model is shown in Figure 11c.

### 3.4. Model Verification

**Figure 12:**
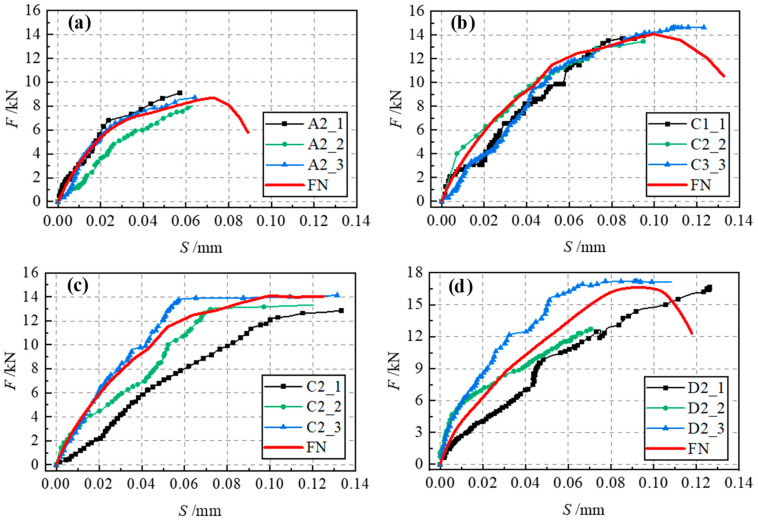
Comparison of experimental and simulated F–S curves (**a**) A2; (**b**) C1; (**c**) C2; (**d**) D2.

The static nonlinear analysis was adopted. Both geometric nonlinearity and material nonlinearity are considered during the analysis. The analysis stops when the load at the loading point drops to 50% of the peak load. Simulations of the interfacial bonding property were completed for specimens A2, C1, C2 and D2. The simulated load–slip curves had a high overall agreement with the experimental curves, as shown in Figure 12. The experimental and simulated values of peak load (*F_p_*) and corresponding slip (*S_p_*) are shown in Table 6. The error between the simulated value and the experimental value was basically kept within 5%. Only the error of the slip in group A2 exceeded 10%. This is attributable to the small bonding length of Group A2. Brittle failure occurred at the peak load, making it difficult to measure the corresponding slip and resulting in a low experimental value. Therefore, the modeling method adopted can simulate the bonding interface successfully.

## 4. Numerical Analysis of Bonding Properties

### 4.1. Parameter Design and Model Establishment

**Figure 13:**
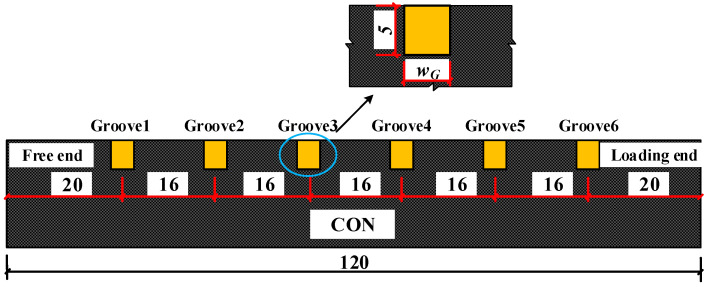
Characteristics of groove (unit: mm).

The parameters of each specimen are shown in Table 7. The bonding interface of the specimens in groups Z, Y and X was Interface I, and that of the specimens in groups W, V and U was Interface II. The characteristics of Interface II are shown in Figure 13. The grooves were arranged from the free end to the loading end. When the number of grooves reached three, no additional grooves were set beyond this point. The constitutive parameters and uniaxial stress–strain curves of concrete with different strength grades were obtained by the method described in Section 3.1. Furthermore, the constitutive parameters are shown in Table 8. The material models of the ECC and HSSWM are consistent with Section 3.1.

### 4.2. Simulation Results and Analysis

#### 4.2.1. Failure Mode

**Figure 14:**
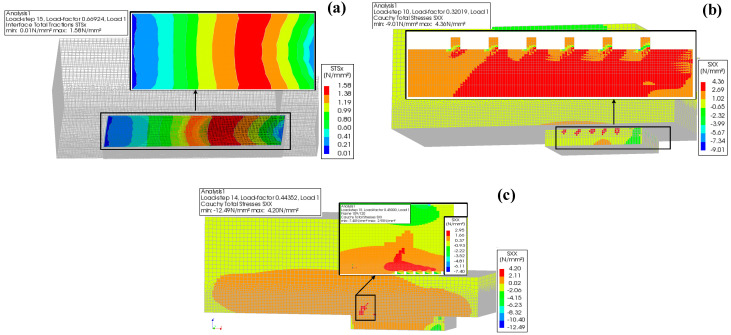
Interfacial stress distribution under peak load: (**a**) Debonding failure of interface; (**b**) ECC extrusion failure; (**c**) Concrete splitting failure.

The interfacial stress distribution under a peak load is shown in Figure 14. Debonding failure of the interface (P) appeared in all the specimens with Interface I. The zero-thickness interface element failed from the loading end. ECC extrusion failure (S1) appeared in all the specimens with Interface II. The ECC was crushed at the grooves from the loading end to the free end. Concrete splitting failure (S2) occurred only in specimen number W1. The concrete strength of W1 was low, leading to stress concentration at the grooves and resulting in concrete splitting.

The failure modes of the simulated specimens were similar to those of the test specimens. Interfacial debonding failure often occurred in specimens with the manual roughening interface. Concrete splitting failure often occurred in the specimens with the grooving interface. However, this is different from the simulation, in that rapture failure often occurred in the test specimens with high-pressure water flushing and grooving interfaces.

#### 4.2.2. Interfacial Load–Slip Curve

**Figure 15:**
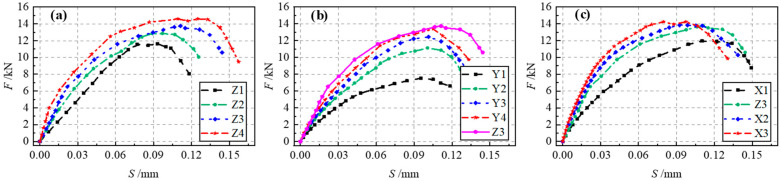
Load–slip curves of specimens with Interface I: (**a**) Group Z; (**b**) Group Y; (**c**) Group X.

**Figure 16:**
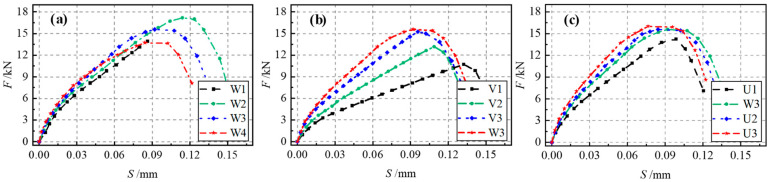
Load–slip curves of specimens with Interface II: (**a**) Group W; (**b**) Group V; (**c**) Group U.

The load–slip curves (F–S curves) of the two types of specimens at the free end were obtained through simulation, as shown in Figure 15 and Figure 16. Meanwhile, the influence of the parameters was further analyzed on the interfacial bonding properties of HSSWM-ECC and concrete. Moreover, the interfacial load–slip curve model of the HSSWM-ECC and concrete was summarized.

As illustrated in Figure 15a, the bearing capacity of Interface I increased with increases in concrete strength. Additionally, the initial slope of the load curve and the slip at peak load increased significantly. This indicates that the bearing capacity and stiffness of Interface I are positively correlated with concrete strength, as a higher concrete strength enhances the mechanical interaction forces at the interface. As can be seen from Figure 15b, the interface bearing capacity first increased, and then remained unchanged as the interfacial bonding length increased. This demonstrates the presence of an effective anchorage length in the specimens with Interface I. When the interfacial bonding length exceeded this effective anchorage length, the interface bearing capacity no longer increased, while the slip at peak load decreased. As displayed in Figure 15c, the bearing capacity of Interface I was not distinctly improved by increasing the interfacial bonding width. The interface width ratio (*b*/*b_c_*) increased simultaneously with the increase in the interface force area and the increase in the interfacial bonding width. The distance between the HSSWM-ECC layer and the concrete edge decreased, which weakened the concrete’s constraining effect on the HSSWM-ECC layer. Consequently, increasing the bonding width reduced the overall stress on the interface. Although the variation in the interface bearing capacity remained minimal, the slip at peak load decreased significantly. This suggests that increasing the interfacial bonding width slightly affects the interface bearing capacity, but notably enhances the interfacial bonding stiffness.

As demonstrated in Figure 16a, the bearing capacity of Interface II first increased and then decreased as the concrete strength was increased. The interface bearing capacity was mainly determined by the strength of the HSSWM-ECC, due to the shear failure of the HSSWM-ECC layer that occurred in most specimens with Interface II. Increasing only the concrete strength reduced the length of the interface engaged in bearing force, leading to a decrease in the interface bearing capacity. Splitting failure first occurred in the concrete of specimen W1 due to its low strength, which limited the effective use of the HSSWM-ECC layer’s properties and resulted in a notably low interface bearing capacity. As shown in Figure 16b, the bearing capacity of Interface II first increased and then remained unchanged as the number of interface grooves increased. The interface bearing capacity stopped increasing once the number of grooves reached a certain threshold, although the corresponding slip continued to decrease. This indicates that increasing the number of grooves can enhance the interface bearing capacity and stiffness, but only within a limited range. As shown in Figure 16c, the bearing capacity of Interface II initially increased and then plateaued as the width of the interface groove was expanded, while the corresponding slip continued to decrease. This effect occurs because increasing the groove width enhances the interface bearing capacity by extending the length of the interface engaged in bearing force. In addition, when the groove width exceeded its depth, the HSSWM-ECC was pulled out at the groove, leading to insufficient utilization of material properties and a decline in the interface bearing capacity.

**Figure 17:**
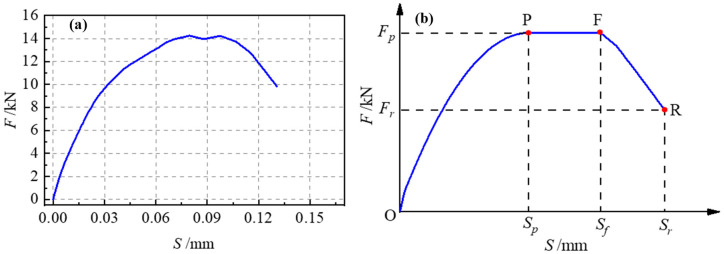
Model of interfacial load–slip curve: (**a**) Typical curve; (**b**) Model of load–slip relationship.

The simulated curves have their horizontal stage and descending stage compared with the test curves. The model of the load–slip relationship was obtained through analysis, as shown in Figure 17. Figure 17a presents the typical load–slip curve of the interface between the HSWM-ECC and concrete. The model was established accordingly, as shown in Figure 17b. The interface load–slip curve of the HSSWM-ECC and concrete can be divided into three stages: the ascending stage, horizontal stage and descending stage. The peak load *F_p_* and corresponding slip *S_p_* for each curve were counted, as shown in Table 9.

Ascending section (OP section): At the initial loading stage, the overall specimen stress was low, and the interface load increased rapidly with slip. As loading continued, the curve displayed noticeable nonlinearity, with the rate of the interface load increase slowing until reaching the peak point, P. This maximum interface load is termed the interface peak load, *F_p_*, and the corresponding slip is termed the peak point slip, *S_p_*.

Horizontal segment (PF segment): After reaching the peak point, the interface load remained basically unchanged with increases in the interface slip, and the horizontal segment appeared at this time. As loading continued, the load–slip curve developed to the failure point, F. The load at this time basically remained unchanged compared with the P point. This load is also termed the interface peak load, *F_p_*, but the corresponding slip changed. This slip is termed *S_f_* at the failure point.

Descending section (FR section): After passing the failure point, the interface load declined with increases in the slip, and the load–slip curve entered the descending stage. When the interface load dropped to 50% of the peak load, it was taken as the failure point R of the specimen. The slip at this time is termed the failure point slip, Sr.

#### 4.2.3. Distribution of Interfacial Stress

The stress distribution curve can be formed by extracting the interface bonding stress (*τ_b_*) along the loading direction under each stage of loads. As a whole, the law of interfacial stress distribution in the simulated specimens was basically same as that obtained in the test. The interfacial stress appeared from the loading end. Then, the stress distribution curve had a peak value and was transmitted to the free end with increases in the load, and the overall curve was convex.

**Figure 18:**
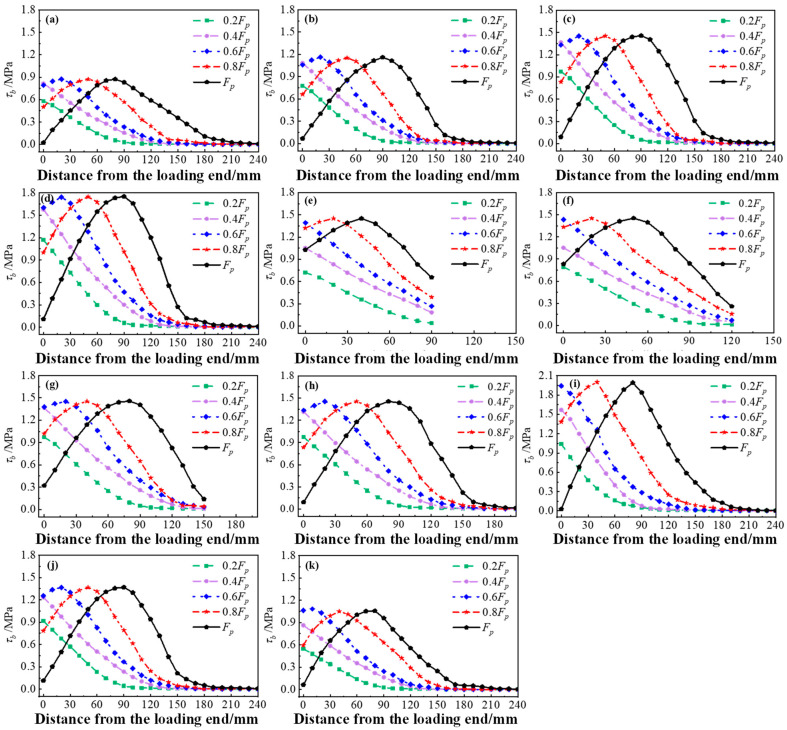
Interfacial stress distribution curves of specimens with Interface I under each load: (**a**) Z1; (**b**) Z2; (**c**) Z3; (**d**) Z4; (**e**) Y1; (**f**) Y2; (**g**) Y3; (**h**) Y4; (**i**) X1; (**j**) X2; (**k**) X3.

The stress distribution and development curves of each group of specimens with Interface I are shown in Figure 18. As illustrated in the curves of Group Z, increasing the concrete strength of the specimens with Interface I improved the interface bond strength. Meanwhile, the distribution range of the interface stress under a peak load gradually decreased, and the length of the interface participating in the force decreased. As displayed in the curves of Group Y, changing the bonding length did not affect the bond strength of the interface under the same bond-slip model. The distribution of the interfacial stress has a limit. Specimens Y1, Y2 and Y3 were all stressed over the whole interface, while specimen Y4 had unstressed sections. This proves that there is an effective anchorage length at the interface between the HSSWM-ECC and concrete. As shown in the curves of Group X, the distribution range of the interface stress remained about 170 mm with increases in the interface bonding width. Meanwhile, the peak interface stress decreased significantly. It can be seen that increasing the interfacial bonding width reduced the interfacial bond strength, but had no effect on the length of the interface participating in the force.

**Figure 19:**
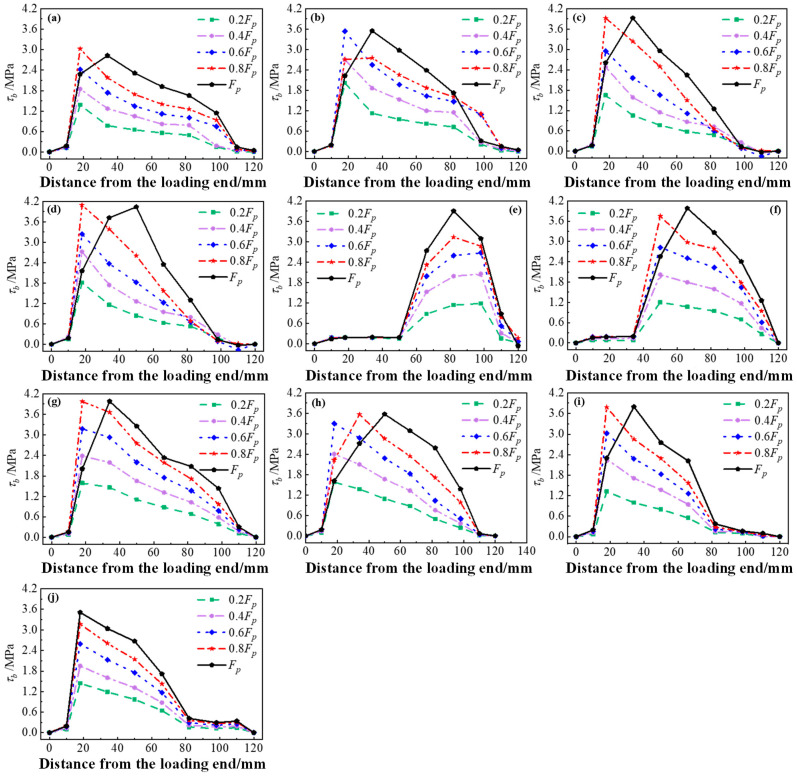
Interfacial stress distribution curves of specimens with Interface II under each load: (**a**) W1; (**b**) W2; (**c**) W3; (**d**) W4; (**e**) V1; (**f**) V2; (**g**) V3; (**h**) U1; (**i**) U2; (**j**) U3.

The interface stress distribution and development curves of each group of specimens with Interface II are shown in Figure 19. As illustrated in the curves of Group W, increasing the concrete strength improved the interfacial bond strength of the specimens with Interface II within a limited range. The reason for this is that the bond strength of the specimens with Interface I was not only affected by the strength of concrete, but also related to the ECC strength. In addition, the distribution range of the interfacial stress gradually decreased with increases in the concrete strength. As displayed in the curves of Group V, the length of the interface participating in the force increased simultaneously with increases in the number of grooves. The interface stress distribution gradually approached the loading end, yet, the peak stress was always around 4.2 MPa. This indicates that increases in the number of grooves had no effect on the interfacial bond strength. The bearing capacity of the interface was mainly improved by increasing the length of the interface participating in the force. As shown in curves of Group U, the interface stress was gradually concentrated towards the loading end with increases in the groove width, while the peak stress remained unchanged. Therefore, changing the groove width had no effect on the bond strength of the interface.

## 5. Theoretical Model

### 5.1. Calculation Model of Effective Anchorage Length

#### 5.1.1. Simulated Value of Effective Anchorage Length

The effective anchorage length was obtained from the interface stress distribution [30]. When the interfacial bonding length exceeds the effective anchorage length, the length of the distribution range of the interfacial bonding stress under peak load is the effective anchorage length. Based on the stress distribution, two effective anchorage length values were obtained for each specimen under the peak load (P point) and failure load (F point).

It is assumed that the bonding strength of Interface I is uniform across all positions. The interfacial bonding force of specimens with Interface II is mainly provided by the mechanical interaction force between the HSSWM-ECC and concrete at the groove, but the contribution from the non-groove is only about 10%. Therefore, the effective anchorage length of Interface II should be the total width of the grooves in this range (*w_GZ_*).

**Figure 20:**
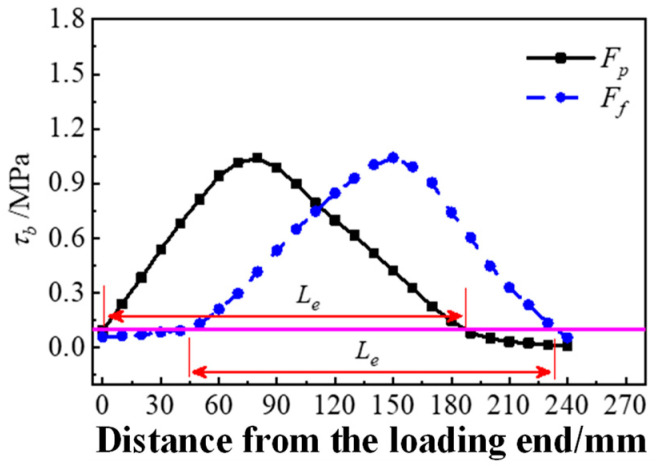
Acquisition of effective anchorage length.

The detailed process for obtaining the effective anchorage length is as follows: First, the interfacial stress distribution curves of each specimen under a peak load (P) and failure load (F) were extracted; then, a horizontal line was made on the stress distribution curve at 10% of the peak bonding stress, and the distance between the two intersection points was the effective anchorage length, as shown in Figure 20. The effective anchorage length of each group of specimens was listed as shown in Table 10, and the average value of the two data was finally used as the effective anchorage length of each specimen.

#### 5.1.2. Calculation Formula of Effective Anchorage Length

The effective anchorage length of the bonding interface between HSSWM-ECC and concrete is affected by the strength of the concrete and the stiffness and thickness of the HSSWM-ECC layer. As illustrated in Table 10, there are obvious differences in effective anchorage lengths between the two interfaces. Thus, different calculation models for effective anchorage length need to be established.

The formula for effective anchorage length proposed by Niedermeier [31] was used for Interface I through analysis and comparison, as shown in Equation (7). The calculated and simulated results are shown in Table 11.

(7) $$L_{e}=\sqrt{\frac{E_{f}t_{f}}{4f_{ct}}}$$

where *L_e_* (mm) is the effective anchorage length; *E_f_* (MPa) is the elastic modulus of the reinforced material; *t_f_* (mm) is the thickness of the reinforced layer; *f_ct_* (MPa) is the tensile strength of the concrete.

It can be seen from Table 11 that the FE simulated values are in agreement with the formula calculated values. The average ratio (F/C) between the simulated and calculated values is 0.979, and the coefficient of variation is 0.01. Therefore, the effective anchorage length of Interface I can be calculated through Equation (8):

(8) $$L_{eH}=\sqrt{\frac{E_{f}t_{f}}{4f_{ct}}}$$

where *L_eH_* (mm) is the effective anchorage length of Interface I; *E_f_* (MPa) is the elastic modulus of the reinforced layer; *t_f_* (mm) is thickness of the ECC; *f_ct_* (MPa) is the tensile strength of the concrete.

The effective anchorage length of Interface II was also related to the concrete strength and the strength and thickness of the ECC. The tensile strength of the concrete and ECC was taken as its characteristic value, referring to the results and analysis in this paper. Furthermore, *L_eG_* = g(*t* × *f_et_*/*f_ct_*) was taken as the objective function to fit the simulated values of Groups W2, W3, W4, U2 and U3. Finally, the expression of *L_eG_* was obtained as shown in Equation (9):

(9) $$L_{eG}=0.88\frac{f_{et}}{f_{ct}}t_{f}$$

where *L_eG_* (mm) is the effective anchorage length of Interface II; *f_et_* (MPa) is the ECC tensile strength; *f_ct_* (MPa) is the tensile strength of the concrete; *t_f_* (mm) is the thickness of the ECC.

### 5.2. Calculation Model of Bearing Capacity

#### 5.2.1. Interface I

It is assumed that the bonding strength of Interface I is equal at any position. A target model of the bearing capacity of Interface I was established by taking tensile strength (*f_ct_*) as the characteristic strength of the concrete, based on numerical analysis and a previous model, as shown in Equation (10):

(10) $$F_{pH}=k_{H}\times \beta_{b}\beta_{l}bL_{eH}f_{ct}$$

where *F_pH_* (kN) is the bearing capacity of Interface I; *b* (mm) is the interfacial bonding width; *L_eH_* (mm) is the effective anchorage length of Interface I; *f_ct_* (MPa) is the tensile strength of the concrete; *β_b_* and *β_l_* are the influence coefficients of the interfacial bonding width and length, respectively. *k_H_* is the comprehensive adjustment coefficient.

Let *F_pH_*_,*b=x*_ = *β_b_*·*F_pH_*_,*b=75mm*_, take *β_b_* = g(*b*/*b_c_*) as the objective function, and fit the coefficient *β_b_* by the specimens in Groups X1, X2, X3 and Z3, as shown in Equation (11). Let *F_pH_*_,*l*_ = *β_l_*·*F_pH_*_,*L_eH_*_, take *β_l_* = g(*l*/*L_e_*) as the objective function, and fit the coefficient *β_l_* by the specimens in Groups Y1, Y2, Y3, Y4 and Z3, as shown in Equation (12).

(11) $$\beta_{b}=\sqrt{\frac{1.2-b/b_{c}}{0.2+b/b_{c}}}$$

where *b* (mm) is the interfacial bonding width; *b_c_* (mm) is the width of the concrete.

(12) $$\beta_{l}={\left(\frac{l}{L_{eH}}\right)}^{2}\left(2.8-1.8\frac{l}{L_{eH}}\right)$$

where *l* (mm) is the interfacial bonding length; *L_eH_* (mm) is the effective anchorage length.

**Figure 21:**
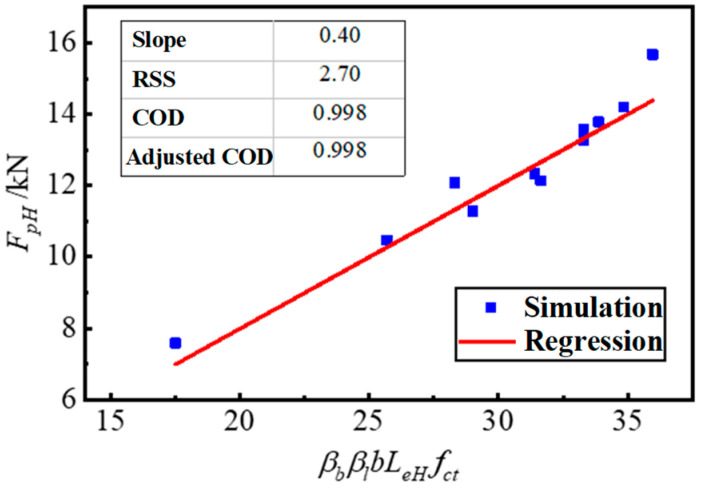
Comprehensive adjustment coefficient KH linear regression analysis.

Linear regression analysis was performed on the data of the fitting group by putting the value of the other parameters into Equation (10). The comprehensive adjustment coefficient *k_H_* = 0.4 was obtained and the COD = 0.998, as shown in Figure 21.

In summary, the bearing capacity calculation model of Interface I between the HSSWM-ECC and concrete was finally obtained as follows:

(13a) $$F_{pH}=0.4\times \beta_{b}\beta_{l}bL_{eH}f_{ct}$$

(13b) $$L_{eH}=\sqrt{\frac{E_{f}t_{f}}{4f_{ct}}}$$

(13c) $$\beta_{l}={\left(\frac{l}{L_{eH}}\right)}^{2}\left(2.8-1.8\frac{l}{L_{eH}}\right)$$

(13d) $$\beta_{b}=\sqrt{\frac{1.2-b/b_{c}}{0.2+b/b_{c}}}$$

where *F_pH_* (kN) is the bearing capacity of Interface I; *β_b_* and *β_l_* are the influence coefficients of the interfacial bonding width and length, respectively; *L_eH_* (mm) is the effective anchorage length of Interface II; *f_ct_* (MPa) is the tensile strength of the concrete; *l* (mm) is the interfacial bonding length; *b* (mm) is the interfacial bonding width; *b_c_* (mm) is the width of the concrete.

#### 5.2.2. Interface II

The bearing capacity of Interface II was mainly provided by the mechanical interaction force at the grooves. There was a wide difference in local forces on the interface. Therefore, the bearing capacity calculation model of Interface II was different from that of Interface I. A target model for calculating the bearing capacity of Interface II was established, combined with numerical analysis and existing research, as shown in Equation (14):

(14) $$F_{pG}=k_{G}\times \eta_{l}bL_{eG}f_{ec}$$

where *F_pG_* (kN) is the bearing capacity of Interface II; *b* (mm) is the interfacial bonding width; *L_eG_* (mm) is the effective anchorage length of Interface II; *f_ec_* (MPa) is the compressive strength of the ECC; *η_l_* is the influence coefficient of the interface anchorage length. *k_G_* is the comprehensive adjustment coefficient.

Let *F_pG_*_,*l*_ = *η_l_*·*F_pG_*_,*L_eG_*_; the influence coefficient *η_l_* of the interface anchorage length was fitted by taking *η_l_* = g(*l*/*L_eG_*) as the objective function and taking the specimens in Groups V1, V2, V3 and W3 as the fitting group, as shown in Equation (15):

(15) $$\eta_{l}={\left(\frac{l}{L_{eG}}\right)}^{2}\left(3.3-2.3\frac{l}{L_{eG}}\right)$$

where *l* (mm) and *L_eG_* (mm) are the interfacial bonding length and the effective anchorage length of Interface II, respectively.

**Figure 22:**
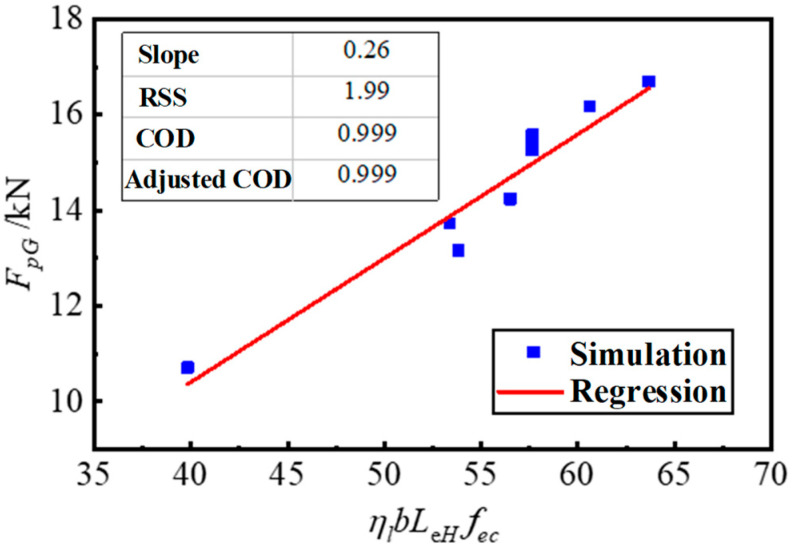
Comprehensive adjustment coefficient *k*_G_ linear regression analysis.

Linear regression analysis was performed on the data of the fitting group by putting the values of the other parameters into Equation (14). The comprehensive adjustment coefficient *k_G_* = 0.26 was obtained and the COD = 0.999, as shown in Figure 22.

In summary, the bearing capacity calculation model of Interface II between the HSSWM-ECC and concrete was finally obtained as follows:

(16a) $$F_{pG}=0.26\times \eta_{l}bL_{eG}f_{ec}$$

(16b) $$\eta_{l}={\left(\frac{l}{L_{eG}}\right)}^{2}\left(3.3-2.3\frac{l}{L_{\mathrm{eG}}}\right)$$

where *F_pG_* (kN) is the bearing capacity of Interface II; *b* (mm) is the interfacial bonding width; *l* (mm) is the interfacial bonding length; *L_eG_* (mm) is the effective anchorage length of Interface II; *f_ec_* (MPa) is the compressive strength of the ECC; *η_l_* is the influence coefficient of the interface anchorage length.

#### 5.2.3. Calculation Model Verification

Specimens were selected from the previous test [25,32] as the verification group, and the calculated values were compared with the experimental values, as shown in Table 12 and Table 13. The ratio between the calculated and experimental values ranged from 0.913 to 1.077, the average was 0.995, and the coefficient of variation was 0.087. This indicates that the calculated values agree well with the experimental values as a whole.

## 6. Conclusions

In this paper, numerical simulation and theoretical analysis of the interfacial bonding properties between HSSWM-ECC and concrete were carried out based on preliminary experimental research, and the following conclusions are drawn:

Three failure modes were found as follows: debonding failure of the interface, ECC extrusion failure at groove and concrete splitting failure. The failure mode was mainly affected by the type of interface. The debonding failure always appeared on the specimens with a roughening interface. The ECC extrusion failure at the groove occurred among the specimens with a grooving interface, except for specimens whose concrete strength was insufficient and concrete splitting failure occurred. The interfacial bearing capacity of the roughening interface mainly depended on the concrete strength and the interfacial bonding length; nevertheless, it was slightly affected by the interfacial bonding width.

The interfacial bearing capacity of the grooving interface is primarily enhanced by increasing the mechanical interaction force between materials. Increasing concrete strength alone, without raising the ECC strength, reduces the interface bearing capacity. Additionally, the bearing capacity of the grooving interface is influenced by the number and width of the grooves. A low concrete strength can lead to concrete splitting failure.

The effective anchorage length at each interface type is inversely related to the concrete strength and directly related to the stiffness and thickness of the HSSWM-ECC. Separate formulas for effective anchorage length were developed for each interface type.

Interfacial bearing capacity models for the two interface types were established, accounting for the concrete strength, bonding length, bonding width and interfacial characteristics. The accuracy of these models was validated by comparing the calculated values with test results.

## Figures and Tables

**Table 1:** Parameters of the specimens.

Specimen	Concrete Strength	Interfacial Bonding Width/mm	Interfacial Bonding Length/mm	Interfacial Treatment Method
A1	C30	75	120	R
A2	C40	75	120	R
A3	C50	75	120	R
B1	C40	60	120	R
B2	C40	90	120	R
C1	C40	75	180	R
C2	C40	75	240	R
D1	C40	75	120	F
D2	C40	75	120	G

**Table 2:** Test results.

Specimen	Processing Mode of Interface	Failure Mode	Peak Load/kN	Slip/mm
A1-1	R	P	7.269	0.0468
A1-2	R	P	8.181	0.0475
A1-3	R	P	7.411	0.0518
A2-1	R	P	9.318	0.068
A2-2	R	P	8.015	0.065
A2-3	R	P	8.848	0.069
A3-1	R	P	12.25	0.071
A3-2	R	P	12.02	0.075
A3-3	R	P	12.40	0.082
B1-1	R	P	9.251	0.077
B1-2	R	P	7.853	0.076
B1-3	R	P	9.732	0.065
B2-1	R	P	9.163	0.029
B2-2	R	P	10.24	0.031
B2-3	R	P	8.582	0.038
C1-1	R	P	14.64	0.123
C1-2	R	P	13.47	0.101
C1-3	R	P	13 93	0.095
C2-1	R	R	14.22	0.137
C2-2	R	R	13.53	0.131
C2-3	R	P	12.90	0.142
D1-1	F	P	16.61	0.108
D1-2	F	R	13.01	0.123
D1-3	F	R	15.76	0.097
D2-1	G	S	17.14	0.108
D2-2	G	S	12.67	0.071
D2-3	G	R	16.65	0.126

**Table 3:** Constitutive parameters of concrete.

Cubic Compressive Strength/MPa	Tensile Strength/MPa	Elastic Modulus/MPa	Shear Modulus/MPa	Poisson’s Ratio
35.31	2.20	31,500	12,600	0.2
46.03	2.51	33,500	13,400	0.2
56.17	2.74	35,500	14,200	0.2

**Table 4:** Constitutive parameters of the ECC.

Compressive Strength/MPa	Tensile Strength/MPa	Elastic Modulus/MPa	Shear Modulus/MPa	Poisson’s Ratio
38.43	3.07	14,500	5800	0.22

**Table 5:** Constitutive parameters of steel wire.

*d*/mm	*E_s_*/MPa	*σ_sp_*/MPa	*ε_sp_*	*a*	*b*	*c*
2.4	130,330	1568.30	3.07%	1.33	−3.66	3.33

**Table 6:** Comparison of peak load and slip between simulated and experimental curves.

Group	A2		C1		C2		D2	
	Test	Simulation	Test	Simulation	Test	Simulation	Test	Simulation
Load/kN	8.73	8.52	14.01	14.11	13.55	14.13	16.75	16.55
Slip/mm	0.067	0.075	0.106	0.095	0.097	0.095	0.094	0.102

**Table 7:** Parameters of the simulated specimens.

Specimen	Concrete Strength	Interfacial Bonding Length/mm	Interfacial Bonding Width/mm	Groove Width/mm	Amount of Groove
Z1	C30	240	75	—	—
Z2	C40	240	75	—	—
Z3	C50	240	75	—	—
Z4	C60	240	75	—	—
Y1	C50	90	75	—	—
Y2	C50	120	75	—	—
Y3	C50	150	75	—	—
Y4	C50	200	75	—	—
X1	C50	240	50	—	—
X2	C50	240	80	—	—
X3	C50	240	100	—	—
W1	C30	120	75	4	6
W2	C40	120	75	4	6
W3	C50	120	75	4	6
W4	C60	120	75	4	6
V1	C50	120	75	4	3
V2	C50	120	75	4	4
V3	C50	120	75	4	5
U1	C50	120	75	3	6
U2	C50	120	75	5	6
U3	C50	120	75	6	6

**Table 8:** Constitutive parameters of concrete.

Strength Grade of Concrete	Cubic Compressive Strength/MPa	Tensile Strength/MPa	Elastic Modulus/MPa	Shear Modulus/MPa	Poisson’s Ratio
C30	30	2.01	30,000	12,000	0.2
C40	40	2.39	32,500	13,000	0.2
C50	50	2.64	34,500	13,800	0.2
C60	60	2.85	36,000	14,400	0.2

**Table 9:** The simulated value of the peak load and corresponding slip.

Specimen	*F_p_*/kN	*S_p_*/mm	Failure Mode
Z1	11.57	0.077	P
Z2	12.86	0.089	P
Z3	13.63	0.106	P
Z4	14.58	0.111	P
Y1	7.53	0.092	P
Y2	11.13	0.096	P
Y3	12.48	0.102	P
Y4	13.41	0.099	P
X1	11.95	0.110	P
X2	13.85	0.093	P
X3	14.25	0.079	P
W1	13.93	0.086	S2
W2	16.72	0.104	S1
W3	15.58	0.092	S1
W4	13.27	0.077	S1
V1	10.71	0.132	S1
V2	13.16	0.109	S1
V3	15.26	0.096	S1
U1	14.27	0.099	S1
U2	15.24	0.078	S1
U3	15.52	0.071	S1

**Table 10:** Effective anchorage length of each specimen.

Specimen	Effective Anchorage Length/mm		
	P Point	F Point	Average
Z1	182.03	193.24	187.64
Z2	166.59	177.96	172.28
Z3	157.24	163.86	160.55
Z4	153.82	152.57	153.20
X1	162.83	165.37	164.10
X2	158.43	165.24	161.84
X3	162.79	163.69	163.24
W2	24	24	24
W3	20	20	20
W4	16	16	16
U2	20	20	20
U3	20	20	20

**Table 11:** The simulated and calculated values for the effective anchorage length.

Specimen	Z1	Z2	Z3	Z4	X1	X2	X3	Average	COV
F/mm	187.64	172.28	160.55	153.2	164.1	161.84	163.24	-	-
C/mm	190.39	174.17	165.72	159.49	165.72	165.72	165.72	-	-
F/C	0.99	0.99	0.97	0.96	0.99	0.98	0.99	0.979	0.01

**Table 12:** Comparison of bearing capacity calculation results of Interface I.

Specimen	Calculation Value/kN	Experimental Value/kN	Ratio
A1	8.21	7.62	1.077
A2	9.27	8.73	1.062
A3	11.71	12.22	0.958
B1	9.01	8.95	1.007
B2	9.85	9.33	1.056
C1	12.80	14.01	0.913
C2	12.80	13.55	0.944
Average	-	-	1.003
COV	-	-	0.065

**Table 13:** Comparison of bearing capacity calculation results of Interface II.

Specimen	Calculation Value/kN	Experimental Value/kN	Ratio
N3	11.56	11.27	1.026
N4	15.15	14.77	1.026
T2	12.12	13.04	0.929
T3	16.93	16.13	1.050
S3	15.11	14.69	1.029
Average	-	-	1.012
COV	-	-	0.047

## Data Availability

The original contributions presented in this study are included in the article. Further inquiries can be directed to the corresponding author.
